# Associated factors for dropout of first versus third doses of pentavalent vaccination in Tanzania

**DOI:** 10.1016/j.vaccine.2025.126962

**Published:** 2025-04-30

**Authors:** Robert Tillya, Gumi Abdallah, Hajirani Msuya, Shraddha Bajaria, Sally Mtenga, Charles Festo, Grace Mhalu, Josephine Shabani, Ibrahim Msuya, William Mwengee, Honorati Masanja, Abdallah Mkopi

**Affiliations:** a Ifakara Health Institute, Dar es Salaam, Tanzania; b Independent Researcher; c World Health Organization, Tanzania Country Office, Dar es Salaam, Tanzania

**Keywords:** Pentavalent, Vaccination, Dropout, Tanzania, Zanzibar, Immunization

## Abstract

The pentavalent is a vaccine against *Diphtheria, Pertussis, Tetanus, Hepatitis B, and Haemophilus type B influenza.* A child is considered a pentavalent vaccination dropout if they have received the first dosage as advised but have not obtained the third dose. In Tanzania, the first-dose receiver of pentavalent was approximately 97 %, whereas only 89 % received a third dose. Unfortunately, no studies have been done in Tanzania to evaluate the factors at the national level that are linked with first-versus third-dose pentavalent vaccine dropout; hence, we explored these factors here for the first time. A cross-sectional survey of randomly selected households was conducted. The sample size was calculated to provide overall, age- and sex-specific coverage estimates for measles-rubella vaccine evaluation among children aged between 9 and 59 months at the national level, as explained elsewhere. The fieldwork activities were done for one month from November to December 2019 for both Zanzibar and Tanzania Mainland. A total of 4460 caregivers of children aged 12–23 months were interviewed for routine immunization services, and a total of 4403 caregivers were included in this analysis of the uptake of the pentavalent vaccine. The number of children who received the first dose of the pentavalent vaccine was 4020 (91.5 %), while the number of children who received the third dose of the pentavalent vaccine was 3915 (89.4 %). The overall pentavalent vaccination dropout rate was 2.3 %. The rate was lower in Zanzibar (0.9 %) than in the Tanzanian mainland (2.4 %). Wealth quintile, sex of caregivers, and education were factors significantly associated with the pentavalent-3 dropout rate among children aged 12–23 months in Tanzania. Our results provide strong support for further efforts to improve current vaccination coverage to optimize the use of prioritized, timely, and appropriate interventions at the regional and district levels and to improve the health education given to expectant women during their clinic visits so they may comprehend the value of routine immunization.

## 1 Introduction

One of the most cost-effective public health initiatives to lower childhood morbidity and mortality is immunization [1]. Immunizations prevent nearly 3 million child deaths annually, attributed to vaccine-preventable diseases (VPD) like measles, diphtheria, tetanus, pertussis (DTP), and influenza [2]. An estimated two million deaths worldwide are attributed to VPDs each year, with over 1.5 million of those deaths happening in children under the age of five, accounting for 15 % of all deaths among this age group [3]. The Central and Southern regions of Sub-Saharan Africa account for about 80 % of child deaths worldwide, which are attributable to incomplete immunization coverage [4].

The recommended times to dose pentavalent 1 to 3 are 6 weeks, 10 weeks, and 14 weeks, respectively [5]. The pentavalent is a vaccine against *Diphtheria, Pertussis, Tetanus, Hepatitis B, and Haemophilus type b influenza* [6,7]. A child is considered a pentavalent vaccination dropout if they have received the first dosage as advised but have not obtained the third dose [8]. Low vaccination dropout rates signify good access to and usage of immunization services performance [9]. In 2019, 90 % of newborns globally received the first dose of the diphtheria-pertussis-tetanus (DPT1) vaccine, while 85 % of newborns globally received three doses of the DPT3 vaccine [10]. The DTP-3 vaccination was not given to 19.7 million newborns globally in 2019, a significant indicator of the ineffectiveness of immunization campaigns [10].

The Global Vaccine Action Plan (GVAP), which uses DPT3 coverage as a benchmark, emphasizes the significance of geographical parity in vaccine coverage by establishing dual targets of 90 % national coverage, and 80 % coverage for all districts within countries by 2020 [11]. Between 2000 and 2016, DPT1 and DPT3 coverage had increased while DPT1–3 dropout rates declined in Africa [12].

According to the Tanzania Demographic and Health Survey (TDHS) 2016, the coverage of the first dose of pentavalent was 97 %, whereas only 89 % received a third dose [13], nationally. According to our review of the literature, no studies have been done in Tanzania to evaluate the factors at the national level, that are linked with first-versus third-dose pentavalent vaccine dropout, hence we explored here for the first time. A study conducted in Gambia showed that, when caregivers attended fewer than four antenatal care sessions, when children had no health card or whose card was lost, and resided in urban areas, there were increased odds of pentavalent dropout [14]. The study conducted by Shrestha, S.R et al., revealed that the dropout of third dose pentavalent vaccine was mainly seen in infants of parents living in rented houses, and according to gender, dropout was high among male infants, busy parents, forgotten date of immunization, visit to other immunization centers and sick infants [15].

This study explored the pentavalent coverage and the factors associated with the pentavalent vaccination dropout rate among children aged 12–23 months in Tanzania.

## 2 Methods

### 2.1 Study design

This cross-sectional survey was conducted among caregivers of children aged 12–23 months from randomly selected households.

#### 2.1.1 Study Settings

The survey was conducted in 31 regions, 26 from Mainland Tanzania and 5 from Zanzibar. The administrative division structure for Tanzania's Mainland starts at the regional level, which covers 4–8 districts, with each district having a population ranging from 150,000–450,000 in rural areas and up to a million in urban areas. Each district is then subdivided into divisions, and a division is subdivided into wards, while the ward is subdivided into villages (rural areas) or streets (urban settings). Furthermore, the village is subdivided into hamlets. In Zanzibar, the administrative division is the same except that the villages or streets are named Shehia.

#### 2.1.2 Sample Size and Sampling Procedures

Data for this article were collected during the survey described in the Mkopi et al. 2021 article [16]. Briefly, the sample size was calculated to provide overall, age- and sex-specific coverage estimates for measles-rubella vaccine evaluation among children aged between 9 and 59 months at the national level as explained elsewhere. In summary, the desired precision of ±5 % with an expected coverage of 95 % was used, 69 enumeration areas were sampled in the first stage using probability proportion to population size from each region yielding a total of 2083 enumeration areas. In the second stage, 7 households with an eligible child were selected within each sampled enumeration area. Thus, a total of 4460 caretakers of children aged 12–23 months were interviewed for routine immunization services, and a total of 4403 caretakers were included in this analysis of the uptake of the pentavalent vaccine.

A two-stage stratified cluster sampling procedure was used during data collection; the first stage involved the selection of enumeration areas (clusters) from the updated list of 2018 provided by the Nation Bureau of Statistics (NBS) using a probability proportional to size (PPS) of the enumeration areas. The second stage involved a simple random selection of seven households from each selected enumeration area (EA). The list of households prepared by village leaders (Tanzania Mainland) and Shehas (Zanzibar) residing in the selected enumeration areas, was used as a sampling frame. Households were defined as persons living together and eating in the same kitchen. Eligible households were defined as having had a child aged 12–23 months. All caregivers aged 18 years and above who were either usual residents or visitors and slept in the selected households the night preceding the survey were eligible for the interview.

#### 2.1.3 Data collection

A total of 171 experienced research assistants (RAs) were recruited from both the Tanzanian mainland and Zanzibar and trained for 3 consecutive days in Dar es Salaam, followed by a one-day pilot and feedback session. The RAs were divided into small groups of 4–10 people depending on workload, with a supervisor for each group to oversee all research fieldwork activities. A quantitative structured questionnaire was used to collect information such as household-level and sociodemographic information and caregivers' perceived reasons for not completing the vaccination schedule. The fieldwork activities were done for one month from November to December 2019 for both Zanzibar and Tanzania Mainland.

#### 2.1.4 Primary outcome and explanatory variables

The primary outcome variable was pentavalent vaccination dropout, defined as children aged 12–23 months who received the first recommended dose of the pentavalent vaccine and missed the third recommended dose of it, as assessed by caregivers' cards and history. In other words, it was a subtracting the number of children who received the third dose of pentavalent vaccine from the number who received the first dose gives the number of dropouts. The number of dropouts was divided by the number of first dose and multiplied by 100 to get the percent dropout. To review a child's vaccination status, the caregiver was required to present a child's clinic card. If the child did not have a clinic card or the caregiver was unable to present the clinic card, the interviewers gathered childhood immunization data for children aged 12 to 23 months based on the caregiver's memory. Thus, both cards and verbal history from caregivers were used to assess the childhood immunization data as recommended by WHO [17]. Explanatory variables were categorized into three levels; household, caregivers, and child. The explanatory variables at the household level were household size (number of persons in the household), household head's occupation, and household wealth quintile. The explanatory variables at the caregiver level were age, sex, education status, and marital status. The explanatory variables at the child level were sex, age, and clinic card.

#### 2.1.5 Data management and analysis

Survey questionnaires were administered in Swahili using the Open Data Kit (ODK) Collect software on the Samsung Galaxy [18]. Data management included data quality checks and friendly error messages as part of the data quality checks. Analysis was done using STATA (version 16; College Station, Texas, USA). The analysis uses survey- weights to adjust for study design and unequal sampling probabilities [19]**.** The dropout rate of the pentavalent vaccine was calculated by (Total number of infants receiving the first dose of the pentavalent vaccine - Total number of infants receiving the third dose of pentavalent vaccine) / Total number of infants receiving the first dose of pentavalent vaccine * 100 [20]. We constructed a social economic status (SES) index with principal component analysis (PCA) [21]. The PCA is a multivariate statistical technique that reduces the number of variables in a data set to a smaller number. The main steps in constructing an SES index in this analysis were the selection of households' assets, application of PCA, interpretation of results, and classification of households into fourth socio-economic groups [22]**.** The household's assets used for SES construction were a bicycle, car, motorcycle, radio, fridge, television, watch, couch, bed, iron, mattress, wardrobe, water pump, sewing machine, satellite dish, fan, and cellphone. Vaccination coverage point estimates and one-sided upper and lower at 95 % confidence bounds were calculated using sample weights and a Taylor series linearization method [17]. The logistic regression model was used to assess factors associated with missing the third of the pentavalent among children aged 12–23 months while controlling for confounding factors such as sex and age. The association between routine immunization coverage and explanatory variables was examined using a logistic regression model. Explanatory variables were included in a multivariable model if the association with the primary outcome had a *p*-value of 0.25 on univariable analysis.

## Ethics statement

Ethical clearance and research permits were obtained from ethical review committees from Ifakara Health Institute's (IHI) Institutional Review Board (IHI-IRB), the National Institute for Medical Research (NIMR), the President's Office Regional Administration and Local Government (PORALG) for Tanzania Mainland, as well as the Zanzibar Medical Research and Ethics Committee (ZAMREC) from Zanzibar. The details of the clearances are further explained in Mkopi et al. 2021 [16]. Individual written informed consent was obtained from the head of the household. The study complied with the International Ethical Guidelines for Biomedical Research Involving Human Subjects [23]**.**

## 3 Results

### 3.1 Characteristics of the survey sample

Caregivers from 14,550 households with 6–59-month-old children were visited in the survey for measles-rubella coverage evaluation [16]. Of these, 4560 households had caregivers of 12–23-month-old children that were included in this analysis. Out of 4560 households, 3604 (79.8 %) had between 1 and 6 members, while 956 households (20.2 %) had 7 or more members. Of these, 2726 (55.8 %) households were involved in subsistence farming, 1142 (27 %) were employed and 692 (17.2 %) were involved in other socio-economic activities. Furthermore, the distribution of households' wealth across groups was very similar, ranging from 18.3 % of them in the lowest quintile and 22.9 % in the highest quintile Table 1. The age distribution of 4461 caregivers was, 1446 (31.5 %) aged between 18 and 24 years, 1957 (43.9 %) had age range 25–34 years, and 1058(24.6) aged 35 years and above. The majority, 4408 (98.6 %) were female caregivers. Of all the caregivers 2861 (61.6 %) had primary education or none while 950 (24.6 %) had acquired secondary education or above. The majority 3782 (84.5 %) of the caregivers were married or cohabited, while 394 (9 %) were divorced/separated, and 216 (4.9 %) caregivers were single. Out of 4403 children, 2230 (51.5 %) were male and 2173 (48.5 %) were female. About half of the children were aged from 12 to 17 months (53.2 %) while 46.8 % were aged from 18 to 23 months.

**Table 1:** Socio-demographic characteristics among caregivers of children aged 12–23 months interviewed for routine immunization services in Tanzania, 2019.

Variable	Weighted % (95 % CI)	n
Household related variables (*N* = 4560)		
Household size (persons)		
1–6	79.8 (78.3–81.2)	3604
7 or more	20.2 (18.8–21.7)	956
Household head's occupation		
Subsistence farming	55.8 (53.8–57.8)	2726
Employed	27 (25.3–28.7)	1142
Other	17.2 (15.7–18.7)	692
Household wealth quintile		
Lowest	18.3 (17.1–19.7)	916
Second	18.7 (17.4–20.1)	894
Middle	19 (17.8–20.4)	897
Fourth	21.1 (19.7–22.5)	942
Highest	22.9 (21.2–24.6)	911
Caregiver-related variables (*N* = 4461)		
Caregiver's age (years)		
18–24	31.5 (30–33.1)	1446
25–34	43.9 (42.3–45.5)	1957
≥ 35	24.6 (23.1–26.1)	1058
Caregiver's sex		
Male	1.4 (0.9–1.9)	53
Female	98.6 (98.1–99)	4408
Caregiver's education		
None and Primary	61.6 (59.8–63.4)	2861
Secondary and above	24.6 (23–26.3)	950
Don't know / Missing	13.8 (12.5–15.1)	650
Caregiver's marital status		
Single	4.9 (4.2–5.7)	216
Widowed	1.6 (1.23–2.2)	69
Divorced/Separated	9 (8–10)	394
Married/Cohabited	84.5 (83.2–85.7)	3782
Child-related variables (*N* = 4403)		
Child's sex		
Male	51.5 (49.9–53.2)	2230
Female	48.5 (46.8–50.1)	2173
Child's age (months)		
12–17 months	53.2 (51.5–54.9)	2332
18–23 months	46.8 (45.1–48.5)	2071

### 3.2 Pentavalent vaccine coverage and pentavalent-3 dropout rate among children aged 12–23 months

Approximately 91.5 % (95 % CI: 90.5–92.5 %) of children nationwide between the ages of 12 and 23 months received their first dose of the pentavalent vaccination. These estimates were slightly higher in Zanzibar at 93.4 % (95 % CI: 87.3–96.7 %) compared to Mainland Tanzania at 91.4 % (95 % CI: 90.3–92.4 %). The pentavalent-1 coverage varied across the regions, from 85.2 % (95 % CI: 79.5–89.5 %) in Tabora to 100 % in Kusini Unguja.

Overall, an estimated 90.7 % (95 % CI: 89.9–91.7 %) of the children aged 12–23 months received the second dose of the pentavalent vaccine. These estimates were slightly higher in Zanzibar at 94.1 % (95 % CI: 87.9–97.2 %) compared to Mainland Tanzania at 90.5 % (95 % CI: 89.3–91.5 %). The pentavalent-2 coverage varied across the regions, from 80.6 % (95 % CI: 74.7–85.4 %) in Tabora to 100 % in Kusini Unguja (Table 2).

**Table 2:** Pentavalent vaccine coverage rate and pentavalent-3 dropout rate among children aged 12–23 months by region in Tanzania, 2019.

Regions	Pentavalent-1		Pentavalent-2		Pentavalent-3		Dropout rate (Pent-1 - Penta-3)	
	Weighted % (95 % CI)	n/N	Weighted % (95 % CI)	n/N	Weighted % (95 % CI)	n/N	Weighted % (95 % CI)	n/N
Overall	**91.5 (90.5–92.5)**	**4020/4403**	**90.7 (89.9–91.7)**	**3975/4403**	**89.4 (88.3–90.5)**	**3915/4403**	**2.3 (1.8–2.8)**	**112/4403**
Tanzania Mainland	**91.4 (90.3–92.4)**	**3889/4263**	**90.5 (89.3–91.5)**	**3843/4263**	**89.1 (88–90.2)**	**3784/4263**	**2.4 (1.9–3)**	**111/4263**
Zanzibar	**93.4 (87.3–96.7)**	**131/140**	**94.1 (87.9–97.2)**	**132/140**	**93.1 (85.7–96.8)**	**131/140**	**0.9 (0.1–6.3)**	**1/140**
								
Kusini Unguja	100	14/14	100	14/14	100	14/14	0	0
Kagera	90.5 (83.9–94.6)	223/244	90.5 (83.9–94.6)	223/244	90.5 (83.9–94.6)	223/244	0	0
Njombe	92.3 (82.1–96.9)	50/56	92.3 (82.1–96.9)	50/56	92.3 (82.1–96.9)	50/56	0	0
Kaskazini Pemba	93.5 (67.7–99)	20/21	93.5 (67.7–99)	20/21	93.5 (67.7–99)	20/21	0	0
Kaskazini Unguja	100	13/13	100	13/13	100	13/13	0	0
Mbeya	92.4 (85–96.3)	177/191	92.4 (85–96.3)	177/191	92.4 (85–96.3)	177/191	0	0
Kusini Pemba	91.8 (58.6–98.9)	15/16	91.8 (58.6–98.9)	15/16	91.8 (58.6–98.9)	15/16	0	0
Katavi	75.7 (56.5–88.2)	33/45	75.7 (56.5–88.2)	33/45	75.7 (56.5–88.2)	33/45	0	0
Dar es Salaam	94.1 (90.9–96.2)	411/436	94.2 (90.9–96.3)	411/436	93.8 (90.6–96)	409/436	0.2 (0.1–0.9)	2/436
Manyara	94 (88.5–97)	134/143	94 (88.5–97)	134/143	93.4 (87.9–96.5)	133/143	0.7 (0.1–4.5)	1/143
Kilimanjaro	81 (72–87.5)	126/154	80.6 (71.7–87.1)	125/154	80.3 (71.6–86.8)	125/154	0.7 (0.1–4.6)	1/154
Pwani	95.8 (88.2–98.6)	76/80	95 (87.5–98.1)	75/80	94.6 (86.9–97.9)	75/80	1.2 (0.2–7.5)	1/80
Kigoma	95.8 (91.9–97.9)	231/242	95.5 (91.6–97.6)	230/242	94.4 (90.3–96.8)	228/242	1.4 (0.4–4.5)	3/242
Mara	92.4 (75.4–90)	178/207	83.4 (74.9–89.4)	177/207	82.5 (74.2–88.6)	175/207	1.5 (0.5–4.5)	3/207
Ruvuma	99.3 (95–99.9)	120/121	99.3 (95–99.9)	120/121	97.5 (92.6–99.2)	118/121	1.8 (0.4–6.8)	2/121
Mjini Magharibi	90.6 (80.1–95.5)	69/76	91.8 (81.4–96.6)	70/76	90 (76.8–96)	69/76	1.9 (0.3–11.5)	1/76
Lindi	96.1 (84.9–99.1)	61/65	96.1 (84.9–99.1)	61/65	94.2 (82.7–98.2)	60/65	1.9 (0.3–12.9)	1/65
Arusha	92 (85.7–95.7)	125/137	92 (85.7–95.7)	125/137	90.1 (83.4–94.2)	123/137	1.9 (0.4–0.9)	2/137
Mtwara	97.4 (93–99.1)	167/179	96.9 (92.5–98.7)	129/134	95.5 (90.4–97.9)	127/134	1.9 (0.6–6.4)	3/134
Songwe	92.9 (83.3–97.1)	64/69	91.9 (82.5–96.5)	63/69	90.9 (81.7–95.8)	62/69	2 (0.5–7.3)	2/69
Tanga	88.3 (82.4–92.4)	147/167	87.5 (80.9–92.1)	146/167	86.9 (90.9–91.3)	145/167	2 (0.6–6.2)	3/167
Geita	94.8 (91.4–97)	225/239	94.1 (90.2–96.5)	223/239	92.8 (88.4–95.7)	220/239	2.4 (1–6.1)	6/239
Shinyanga	96 (91.7–98.2)	144/150	94.7 (89.2–97.4)	142/150	93.1 (86.5–96.6)	140/150	3 (0.9–8.8)	4/150
Dodoma	91 (83.6–95.2)	145/159	89.6 (81–94.6)	143/159	88.5 (80.4–93.6)	142/159	3.2 (1.1–8.6)	4/159
Morogoro	91.8 (85.6–95.5)	167/179	91.1 (84.9–94.9)	165/179	88.2 (81.7–92.6)	161/179	3.7 (1.6–8)	6/179
Iringa	93.1 (82.6–97.5)	78/85	91.3 (81.5–96.2)	76/85	89 (79.2–94.6)	73/85	4.1 (1.7–9.5)	5/85
Mwanza	93.6 (89.6–96.1)	270/290	91.7 (87.5–94.6)	265/290	89.7 (84.7–93.2)	260/290	4.2 (2–8.4)	11/290
Simiyu	87.3 (81–91.8)	205/234	86.3 (79.6–91)	202/234	83.5 (76.7–88.6)	196/234	4.3 (2.2–8.3)	10/234
Singida	90.9 (82.7–95.4)	101/110	86.7 (78.9–91.9)	96/110	86.2 (78.1–91.6)	95/110	4.7 (1.9–11.3)	6/110
Rukwa	75.3 (61.2–85.4)	55/76	69.2 (54.2–81.1)	51/76	68.1 (53.2–79.9)	50/76	7.2 (2.9–16.4)	5/76
Tabora	85.2 (79.5–89.5)	213/250	80.6 (74.7–85.4)	201/250	74.4 (67.9–80)	184/250	11.2 (7.4–16.7)	30/250

At the national level, the pentavalent vaccination was administered to an estimated 89.4 % (95 % CI: 88.3–90.5 %) of children between the ages of 12 and 23 months. These estimates were slightly higher in Zanzibar 93.1 % (95 % CI: 85.7–96.8 %) compared to Mainland Tanzania 89.1 % (95 % CI: 88–90.2 %). The pentavalent-3 coverage varied across the regions, from 74.4 % (95 % CI: 67.9–80 %) in Tabora to 100 % in Kusini Unguja (Table 2).

Nationally, an estimated 2.3 % (95 % CI: 1.8–2.8 %) of the children aged 12–23 months dropped out of the third dose of the pentavalent vaccine. These estimates were slightly lower in Zanzibar at 0.9 % (95 % CI: 0.1–6.3 %) compared to Mainland Tanzania at 2.4 % (95 % CI: 1.9–6.3 %). The pentavalent vaccination dropout rate varied across the regions, from 0 % to 11.2 % (95 % CI: 7.4–16.7 %) (Fig. 1D). Out of 31 regions, 8 (Kusini Unguja, Kagera, Njombe, Kaskazini Pemba, Kaskazini Unguja, Mbeya, Kusini Pemba and Katavi) had no dropout (Table 2).

Tanzania regions are classified into 9 zones: 1. eastern zone (Morogoro, Pwani, and Dar es Salaam) 2. northern zone (Arusha, Kilimanjaro, and Tanga); 3. lake zone (Kagera, Mwanza, and Mara); 4. western zone (Kigoma, Tabora, and Shinyanga); 5. the central zone (Dodoma, Manyara, and Singida); 6. southwest highlands (Katavi, Mbeya, and Rukwa); 7. southern highlands Zone (Iringa, Njombe, and Ruvuma); 8. southern zone (Lindi and Mtwara) and 9. Zanzibar zone. Pentavalent 1–3 coverage rates ranged from 95.1 % to 100 % in the southern, eastern, and Zanzibar zones (Fig. 1A, Fig. 1B, and Fig. 1C). The same coverage rates were in Ruvuma, Kigoma, and Shinyanga. Related to the pentavalent vaccination dropout, the western zone's Tabora region had a high dropout rate of over 10 %. The regions of Simiyu, Mwanza, Singida, Iringa, and Rukwa had dropout rates, ranging from 5 % to 9 % (Fig. 1D).

**Fig. 1A:**
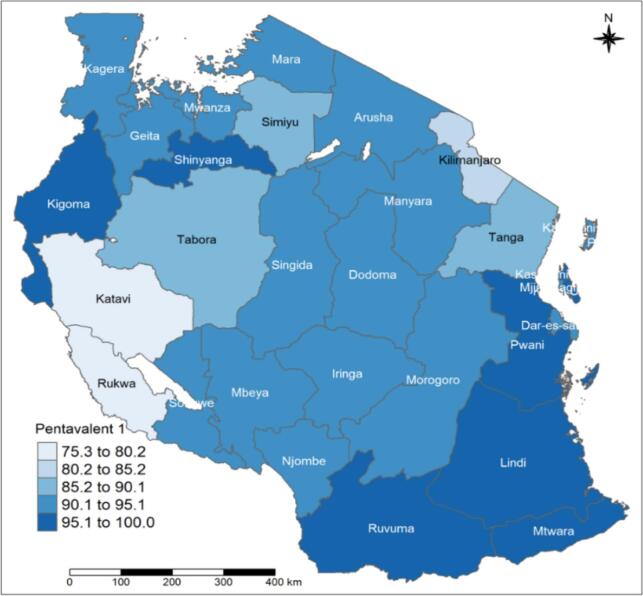
Pentavalent-1 vaccination coverage rate among children with 12–23 months by regions.

**Fig. 1B:**
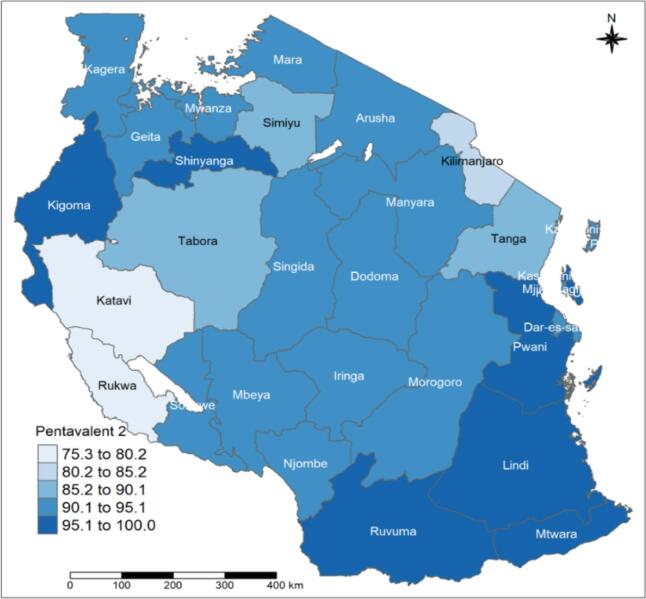
Pentavalent-2 vaccination coverage rate among children with 12–23 months by regions.

**Fig. 1C:**
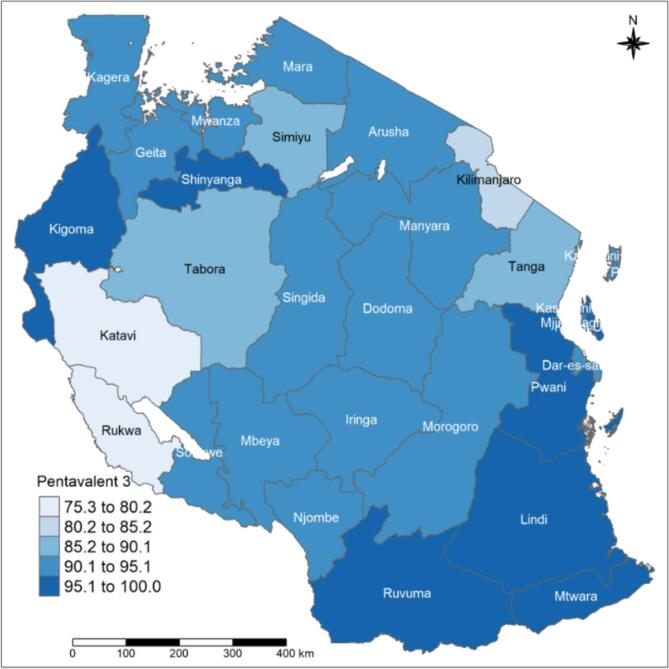
Pentavalent-3 vaccination coverage rate among children with 12–23 months by regions.

**Fig. 1D:**
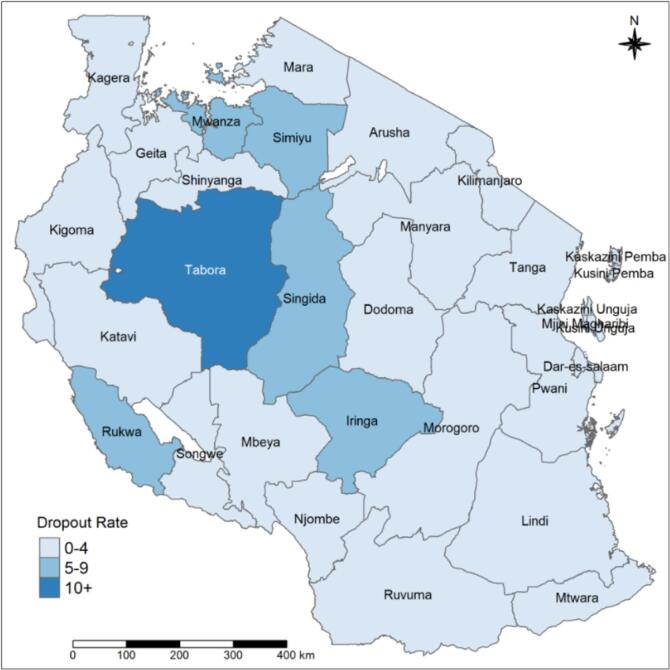
Pentavalent vaccination dropout rate among children with 12–23 months by regions.

### 3.3 Factors associated with pentavalent-3 dropout rate among children aged 12–23 months

Three factors were significantly associated with the pentavalent vaccination dropout rate among children aged 12–23 months in Tanzania. One factor at the household level was the household wealth quintile. In multivariate analysis, children living in households with the middle (AOR: 0.47; 95 % CI: 0.25–0.86; *p* = 0.016), fourth (AOR: 0.46; 95 % CI: 0.25–0.85; *p* = 0.013), and highest wealth quintile (AOR: 0.27; 95 % CI: 0.13–0.53; *p* < 0.001) were less likely to dropout from pentavalent vaccination compared to children living in a household with the lowest wealth quintile. Two factors at the caregiver's level were the caregiver's sex and education. In univariate analysis, children of female caregivers had a 2 % less likelihood to drop out of the pentavalent vaccine compared to those of male caregivers (AOR: 0.02; 95 % CI: 0.019–0.03; p < 0.001). In multivariate analysis, children of caregivers who attended school were significantly less likely to drop out in the pentavalent vaccine compared to those of caregivers who did not attend school (AOR: 0.47; 95 % CI: 0.29–0.77; p < 0.001) (Table 3).

**Table 3:** Crude and adjusted odds ratios for factors associated with pentavalent-3 dropout rate among children aged 12–23 months in Tanzania, 2019.

	Dropout rate		Univariate analysis			Multivariate analysis		

**At the household level (N = 4560)**	**YES**	**NO**	**OR**	**95 % CI**	**p-value**	**AOR**	**95 % CI**	**p-value**
Household size (persons)								
1–6	87	3517	1	**–**	**–**			
7 or more	25	931	0.98	0.59–1.64	0.950			
Household wealth quintile								
Lowest	40	876	1	**–**	**–**	1	**–**	**–**
Second	23	871	0.69	0.38–1.25	0.215	0.69	0.38–1.25	0.215
Middle	17	880	0.47	0.25–0.86	0.016	0.47	0.25–0.86	0.016
Fourth	19	923	0.46	0.25–0.85	0.013	0.46	0.25–0.85	0.013
Highest	13	898	0.27	0.13–0.53	<0.001	0.27	0.13–0.53	<0.001
Household head's occupation								
Other occupation	16	676	1	**–**	**–**			
Subsistence farming	74	2652	1.56	0.87–2.81	0.134			
Employed	22	1120	1.05	0.51–2.16	0.892			
**At the caregiver level (N = 4461)**	**YES**	**NO**	**OR**	**95 % CI**	**p-value**	**AOR**	**95 % CI**	**p-value**
Caregiver's age (years)								
18–24	39	1407	1	**–**	**–**	1	**–**	**–**
25–34	41	1616	0.74	0.46–1.20	0.225	0.73	0.45–1.19	0.205
≥ 35	33	1025	1.11	0.67–1.84	0.673	1.27	0.98–1.64	0.836
Caregiver's sex								
Male	0	53	1	**–**	**–**			
Female	113	4295	0.02	0.01–0.03	<0.001		Omitted	
Caregiver's marital status								
Single	5	211	1	**–**	**–**			
Widowed	2	67	0.76	0.14–4.01	0.749			
Divorced/Separated	9	385	0.92	0.29–2.93	0.885			
Married/Cohabited	97	3685	0.89	0.36–2.19	0.807			
Caregiver's education								
Not attended School	26	608	1	**–**	**–**	1	**–**	**–**
Attended school	87	3740	0.46	0.28–0.76	0.002	0.47	0.29–0.77	< 0.001
**At the child level (N = 4403)**	**YES**	**NO**	**OR**	**95 % CI**	**p-value**	**AOR**	**95 % CI**	**p-value**
Child's sex								
Male	53	2177	1	**–**	**–**	1	**–**	**–**
Female	59	2114	1.07	0.72–1.59	0.735	0.89	0.55–1.42	0.616
Child's age (months)								
12–17 months	64	2268	1	**–**	**–**	1	**–**	**–**
18–23 months	48	2023	0.92	0.61–1.39	0.699	0.84	0.51–1.36	0.475
Child's clinic card								
NO	0	667		Omitted				
YES	112	3624						

## 4 Discussion

We revealed that in 2019, the overall pentavalent vaccination dropout rate was 2.3 % which is significantly lower than the 8.0 % dropout rate reported by the TDHS in 2015 [13]. The overall lower dropout rate indicates a significant improvement in immunization coverage in Tanzania. The DHS survey of data from 33 sub-Saharan African countries, which was conducted between 2010 and 2020 using a multilevel mixed-effect analysis showed that pentavalent vaccination dropout was 20.9 % [24]. The 2.3 % dropout rate is less than the 20.9 % overall pentavalent vaccine dropout rate. The potential explanation for these discrepancies may be due to differences in aggregate data used in Sub-Saharan African countries, while this study used individual country data of Tanzania. Thus, the aggregated data results may show the highest results of pentavalent vaccination dropout rates compared to the individual data in Tanzania. However, some of the Sub-Saharan countries used individual data but still had higher pentavalent vaccination dropout rates than Tanzania. The countries were; Gambia (4.3 %) [14], West Africa (16.3 %) [25], and Ethiopia (17 %) [26]. However, this suggests that Tanzania's stated overall low dropout rate of 2.3 %, reflects high access to and use of immunization services. There were notable regional differences in the pentavalent vaccination dropout rates among 12–23-month-old children in Tanzania. Eight regions were reported to have no pentavalent vaccination dropouts. In contrast, the dropout rates in the remaining 21 regions, range from 0.2 % to 11.2 %. The World Health Organization (WHO) states that a dropout rate of more than 10 % indicates that many people do not use the services [27]. The pentavalent dropout rate in the Tabora region is the highest at 11.2 %. There is a chance that children in the Tabora region could potentially contract VPDs.

In our study, there were factors associated with the pentavalent vaccination dropout rate among children aged 12–23 months in Tanzania. We found that children living in the household with the higher (middle, fourth, and highest) wealth quintiles, were less likely to drop out of the pentavalent vaccination compared to children living in a household with the lowest wealth quintile. Similar findings indicated that household wealth is a major factor in the utilization of vaccination services [28,29]. We also found that children of female caregivers were less likely to drop out of the pentavalent-3 vaccine compared to those of male caregivers. However, the conducted in Ethiopia showed that caregivers who were less gender-empowered had increased odds of pentavalent vaccination dropout [30]. Our study also found that children of caregivers who attended school were less likely to drop out in the pentavalent vaccination compared to those of caregivers who did not attend school. This finding was consistent with studies conducted in Kenya [31], Nepal [15], and Ethiopia [32]. The reason behind this could be that caregivers with higher educational levels probably can understand the importance of child vaccination than caregivers with lower education levels.

The associated factors elucidated the reasons why pentavalent vaccination dropout occurs. To achieve a pentavalent vaccination dropout of less than 10 % in all regions, it is necessary to improve immunization access and utilization. Therefore, it is necessary to enhance programmatic monitoring in vaccine deployment and to improve the activities of immunization campaigns and communication strategies in parts of Tanzania and other sub-Saharan African nations where the pentavalent vaccination dropout rate is more than 10 %.

The present study's limitation is that, in the absence of vaccination records, the caregivers' recall was used to compile data on children's immunization in this national survey. The vaccine coverage rate may be overestimated or underestimated as a result of the caregivers' poor recall. No independent mapping was done, which might likely introduce selection bias among the caregivers interviewed.

In conclusion, our study indicates that the overall pentavalent vaccination dropout was less than 10 % which met targets set by the WHO. However, variation in individual regions still exists as the study found that, one of the regions, had more than 10 % of the pentavalent vaccination dropout. Our results provide strong evidence for further efforts to improve current vaccination coverage and to optimize the use of the following recommendation interventions: 1) To prioritize timely and appropriate interventions at the regional and district levels, it is necessary to analyze the data already available from immunization program to track the development of vaccine coverage. 2) The Ministry of Health must improve the health education given to expectant women during their clinic visits so they may comprehend the value of routine immunization.

## Funding sources

This work was supported by the WHO country office, in Dar es Salaam, Tanzania.

## CRediT authorship contribution statement

**Robert Tillya:** Writing – review & editing, Writing – original draft, Investigation. **Gumi Abdallah:** Writing – review & editing. **Hajirani Msuya:** Writing – original draft. **Shraddha Bajaria:** Writing – review & editing. **Sally Mtenga:** Writing – review & editing. **Charles Festo:** Writing – review & editing. **Grace Mhalu:** Writing – review & editing. **Josephine Shabani:** Writing – review & editing. **Ibrahim Msuya:** Writing – review & editing, Visualization. **William Mwengee:** Writing – review & editing. **Honorati Masanja:** Writing – review & editing. **Abdallah Mkopi:** Writing – review & editing, Writing – original draft, Project administration, Methodology, Investigation, Formal analysis, Conceptualization.

## Declaration of competing interest

The authors declare that they have no known competing financial interests or personal relationships that could have appeared to influence the work reported in this paper.

## Data Availability

Data will be made available on request.
